# Understanding trauma-impaired executive function and its impacts on homeless adults in the UK: a realist evaluative synthesis protocol

**DOI:** 10.1136/bmjopen-2024-093529

**Published:** 2025-06-20

**Authors:** Christina Cooper, Monique Lhussier

**Affiliations:** 1 School of Health, Education and Community Studies, Northumbria University, Newcastle Upon Tyne, UK

**Keywords:** Cognition, Community Participation, Health Equity, Health Services, Neurobiology, Psychosocial Intervention

## Abstract

**Abstract:**

**Introduction:**

Approximately 90% of people with experience of homelessness report adverse childhood experiences, having far-reaching consequences across the life course. Trauma-informed approaches have burgeoned in the last decade; however, biological understandings, including neurological perspectives of the impact of trauma, are typically overlooked. At present, there is little evidence exploring the impacts of executive function (EF) deficits in adulthood as a result of childhood trauma from a lived experience perspective, with none specifically exploring the role these deficits might play in homelessness.

**Methods and analysis:**

The proposed research takes a realist evaluative synthesis approach combining evidence from the extant literature with qualitative data from professionals involved in the delivery of services to support people with experience of homelessness (n=15–20) and people with experience of homelessness who have previously engaged with services (n=15–20).

A combination of keywords (Adverse childhood experiences, executive function, social functioning and complex needs) and their synonyms will be used to search databases MEDLINE, PubMed, SCOPUS, Web of Science and CINAHL. Data analysis will follow a realist logic, developing and refining programme theories. An iterative and cyclical approach to data analysis and evidence synthesis will be taken until the aims of the research have been met.

**Ethics and dissemination:**

Findings from this study will contribute to new understandings of the pathways into and out of homelessness through the lens of EF. Findings will contribute to the development of a trauma-informed care toolkit for service providers. Findings will be disseminated via coproductive workshops, conferences and peer-reviewed publications.

This study has received ethical approval from the ethics committee at Northumbria University.

**STRENGTHS AND LIMITATIONS OF THIS STUDY**

- This study takes a novel approach to understanding the impacts of adverse childhood experiences (ACEs) on pathways into and out of homelessness using a biopsychosocial model to explore the role of executive function impairment.
- Adopting an understanding of causation that is generative, rather than successive, this study aims to bring together concepts of ACEs, executive functioning and homelessness together in explanatory categories as yet unexplored in the literature.
- Applying a realist evaluative synthesis approach facilitates an in-depth exploration of these concepts, combining evidence from the literature with a range of lived and professional experiences.
- This study takes a qualitative approach, which seeks to understand personal experiences and provides no proof of executive dysfunction as a result of trauma beyond what is already available in the literature.
- This study is being conducted, in the first instance, in the Northeast and Cumbria, and therefore findings may be somewhat region specific.

## Introduction

 People experiencing chronic homelessness (PEH), defined as either lengthy (more than 12 months) or repeated (more than four episodes in the last 3 years totalling a minimum of 12 months), face significant inequalities and poorer health outcomes.^1 2^ PEH often have a constellation of intersecting needs, such as poor physical and mental health, addiction and experience of the criminal justice system, which they experience earlier than others.^3^

PEH face significant challenges in accessing and engaging with appropriate services,^4^ contributing to repeated homelessness and a revolving door through which they face repeated needs assessments without receiving appropriate support. The longer this cycle persists, the greater the damage to health.^5^ Poor engagement with services that may contribute to routes out of homelessness has frequently been attributed by professionals delivering care to ‘chaotic lifestyles’ in which health needs are not prioritised by individuals, who may fail to attend appointments and find themselves frequently excluded from services.^6^ However, PEH identified individual factors, including multiple diagnoses, cultural beliefs, trust, stigma and shame, and structural factors, such as financial barriers, long waiting lists and target-driven services, as key barriers to access^7^ which were compounded by the impact of trauma.

Trauma-informed care (TIC) has been widely adopted to support people with complex needs, increasing service engagement and improving outcomes.^8^ TIC is a social movement, driven by a paradigm shift from medical understandings of trauma predominantly focused on physical injury, to models which recognise the role of psychological and social understandings of trauma. Six key principles of TIC have been outlined: safety, trust and transparency, peer support, collaboration and mutuality, empowerment (voice and choice), and individual factors (histories, gender, culture).^8^ However, this move away from the medical model has led to gaps in knowledge around the biological impacts of adverse childhood experiences (ACEs) and the lifelong effects they can have. In this project, we combine evidence from neuroscientific literature, which explores these impairments with qualitative data from PEH exploring lived experiences using a biopsychosocial lens.

Approximately 90% of PEH have had at least one ACE, with over 50% having experienced four or more, compared with 39% and 5%, respectively, in the general population.^9^ ACEs encompass a range of repeated traumatic experiences or chronic stressors occurring under the age of 18 years,^9^ typically classified into 10 categories: physical, emotional, and sexual abuse; physical and emotional neglect; witnessing domestic violence, having a family member affected by mental illness, substance abuse, or incarceration; and losing a parent through separation, divorce or death.^10^ ACEs, along with primary attachment deprivation, have been shown to have significant biopsychosocial consequences across the life course.^11^,^15^

When faced with a stressful situation, activation of the sympathetic nervous system and hypothalamic–pituitary–adrenal axis triggers the release of cortisol and production of glucose in preparation for overcoming or escaping the threat (fight or flight).^16^ Following a stressful experience, there is usually a period of recovery, with support for children typically coming from a parent or caregiver for soothing and building resilience. Where stress is chronic, and support from primary caregivers is not available, these changes can become a permanent part of biological processes, and allostatic overload can occur, impacting the neurological, endocrine and immune systems.^16^ Neuroscientific evidence shows ACEs impact on several brain systems, producing long-lasting changes in brain structure and function.^15 17^ Documented brain changes include reduced global volume; alterations in limbic circuitry, particularly the amygdala and hippocampus, responsible for emotion regulation; and alterations in prefrontal cortex development responsible for executive function (EF).^18^

EF is broadly defined as a set of separate but interacting top-down processes involved in goal-oriented thoughts and behaviours,^19^ particularly useful when undertaking goals in direct conflict with habitualised behaviours.^20^ EF such as inhibition control (self-regulation, emotion control), working memory and cognitive flexibility (attention, mental shifting) drive goal-oriented cognitive capabilities such as reasoning, planning and problem-solving.^18 21^

The impact of trauma on EF was further confirmed in a recent study using brain imaging, which identifies the specific EF impacted, including memory, processing (affective, social, mentalising), problem-solving, decision-making, learning, motivation and self-focus.^22^ Research around trauma and EF is largely quantitative, employing experimental techniques to understand which specific functions have been impaired and the role they play within specific aspects of health or social functioning.^18 21^ However, impacts tend to be considered in a siloed way, with evidence considering discrete areas such as relationship development and maintenance,^23^ school readiness and academic achievement,^24 25^ employment and job success,^26^ mental health,^16^ suicidality^27^ and substance use.^28^ Knowledge of how these issues are interconnected, how they are experienced qualitatively or how they contribute to routes in and out of homelessness is currently lacking. This study is therefore the first to premise, and seek to evidence, a complex, generative causal chain among ACEs, EF, homelessness and access and engagement with services.

## Methods and analysis

### Aim and objectives

#### Aim

The proposed study aims to understand the impact of trauma, and more specifically EF impairments, on people’s day-to-day lives, their homelessness and engagement with support. Findings from this study will inform the continued development of trauma-informed services for PEH.

#### Objectives

The objectives are:

1. To understand the life course experiences of PEH through listening to and interpreting life stories with a trauma-informed lens.
2. To understand how lived experiences may be explained by impaired EF.
3. To understand how these experiences may intersect in cumulative and cyclical ways across the life course.
4. To identify challenges resulting from experiences of EF impairments, which may act as a barrier to engagement with support services from the perspective of PEH.
5. To explore homelessness service providers’ understanding of EF and its relationship to ACEs.
6. To identify current approaches to TIC being implemented in services for PEH.

### Research approach

This study will adopt a realist methodological perspective, which involves identifying generative causal mechanisms and exploring how they work, and under what conditions they work best.^29^ In this study, EF will be considered as a putative underlying mechanism to explain experiences of homelessness and engagement with services.

An evaluative realist synthesis,^30^,^32^ combining methods of realist synthesis with primary qualitative data from key stakeholders, will be employed. As is customary in realist approaches, explanatory statements linking the various aspects of homelessness exposed above will be first developed in initial programme theories (IPTs), which are then refined and tested through primary and published data into finalised programme theories (PTs). These are formulated using the well-recognised realist heuristic of context+mechanism=outcome.^33^ In realist thinking, PTs are used as the unit of analysis, bringing together various sources of evidence. Realist methodology lends itself ideally to study the kind of complex issues that we are focussing on, such as ACEs, EF and homelessness, which are each represented by distinct fields of scholarly endeavour, but for which the overlaps and interactions have never yet been examined.

The study will be informed and guided by six lived experience experts (Lexperts).

### Patient and public involvement

This study has been developed in consultation with eight Lexperts (all men, aged 30–56 years old), drawing on their experiences of childhood trauma and the impacts it has had throughout their lives. They have advised that the research should centre the stories and experiences of people with lived experience of these impacts. Lexperts also felt it was important to better understand how trauma affects engagement with services and to explore aspects that current trauma-informed approaches fail to address. To meet these specific objectives, a qualitative approach to the work was agreed.

Lexperts will continue involvement throughout the research, including development and piloting of research materials, recruitment support, interviews with professionals, data analysis and interpretation, and dissemination of findings. Lexperts will be recruited from partner organisations involved with the broader research project. Equality, diversity and inclusion will be considered during the recruitment process. We work flexibly with our Lexperts and do not expect commitment over the full length of the project, meaning we will not over-rely on the same individual or group of individuals as this could result in undue pressure or stress negatively impacting health and well-being. Lexperts will be supported by the project lived experience coordinator who has over 10 years’ experience of working with and supporting people with lived experience of homelessness and/or multiple and complex needs, as well as by the university team. This will ensure that, while the project centres on traumatic experience, it does not, in and of itself, cause retraumatisation. The project will follow thorough safeguarding policies, ensuring the well-being of our Lexperts throughout the project.

### Realist review of existing evidence

The proposed realist review applies the Realist and Meta-narrative Evidence Syntheses: Evolving Standards publication guidelines,^34^ including the following four iterative steps:

1. Research question development.
2. IPT and search strategy development.
3. Study selection and appraisal.
4. Data extraction, analysis and synthesis.

### Research question development

I can recall the abuse from years ago, in fact it is my earliest memory, in vivid detail but if you were to ask me what I was doing last Tuesday I couldn’t tell you (K, 55).

This quote formed part of K’s narrative in recounting his pathway into and experiences of homelessness. Drawing on the narratives from K and the other Lexperts, we began to consider the impact of trauma on working memory and broader executive functioning, and the role this might play in pathways into, experiences of and routes out of homelessness, including engagement with support services. Combining thinking from two workshop discussions and one Lexpert consultation panel (each lasting 1–2 hours) with existing knowledge of psychological constructs of well-being, the research question below was developed:

Does EF impairment resulting from trauma impact on social functioning and/or the development of complex health and social care needs, contributing to pathways into and difficulties escaping from chronic homelessness?

### IPT and search strategy development

Based on input from Lexperts, a broad IPT was developed:

In PEH, ACEs (context), lead to EF impairments (mechanism) impacting social functioning and the development or worsening of multiple and complex needs, contributing to pathways into homelessness, challenges in engaging with services designed to help and poor health and well-being outcomes (outcome).

It is anticipated that under this broad IPT, a multitude of more specific IPTs will be developed, around EF as an underpinning mechanism for experiences of homelessness. A scoping review was undertaken to ensure that there was sufficient breadth and depth of literature to undertake the review, to identify search terms and core themes which would form the basis of the PTs, returning 46 relevant papers.

Search terms for this used key words from the IPT and relevant synonyms, such as: ‘Trauma and brain function’; ‘Trauma and executive function/dysfunction’; ‘Abuse and executive function/dysfunction’; ‘Adverse childhood experiences and executive function/dysfunction’; ‘Adverse childhood experiences and cognitive impairments’. Initially, each of these search terms was also combined with homelessness; however, no literature was available, reinforcing the need for this study. Evidence was then screened for data relating to social functioning (relationships, education, employment), complex needs (substance use, mental health and suicidality), in line with the research question.

Search strategy: a combination of keywords will be used to search databases MEDLINE, PubMed, SCOPUS, Web of Science and CINAHL. A range of search terms will be used to include both MeSH terms and additional key terms where no MeSH terms have been identified. The main search terms used include: Adverse childhood experiences, ACEs, childhood trauma, traumatic childhood experiences, experiences of early abuse, executive function, executive control, cognition, cognitive control, relationships, interpersonal relationships, family, education, schooling, educational attainment, educational achievement, employment, employment status, occupational status, work, career, unemployment, mental health, mental ill health, mental illness, poor mental health, suicide, suicidal ideation, attempted suicide, substance use, drug use, substance/drug abuse, dependence, addiction, alcohol use, alcohol abuse, alcohol dependence and alcoholism. Advanced search options will be used to combine search terms and their truncations in every database. Further search terms may be identified throughout the iterative research process.

The following algorithm will be used to conduct database searching: Adverse childhood experiences [OR variations} AND Executive function [OR variations] AND one of the following six factors: Relationships [OR variations}, Education [OR variations], Employment [OR variations], Mental health [OR variations], Substance use [OR variations], Suicide [OR variations].

A full list of search terms will be included on publication of the study findings.

In addition to this, we will use search strategies such as citation tracing, kinship and sibling papers and snowballing to capture all data relevant to the developing PTs. Further additional searches may be undertaken throughout the duration of the study in response to new and emerging theories.

### Study selection and appraisal

Documents identified in the previous step will be screened and selected for inclusion using a three-step process. Documents will be screened initially for relevance using title and abstract screening. Following this, those identified for possible inclusion will undergo a full-text screening against inclusion and exclusion criteria (see table 1).

**Table 1:** Inclusion and exclusion criteria

Inclusion criteria	Exclusion criteria
Focus on EF Focusing on adults Focus on health and/or social functioning No geographical restriction Language English Qualitative and quantitative evidence	No focus on EF Focusing exclusively on children No focus on health or social functioning Published in a language other than English

EF, executive function.

Documents that meet the inclusion criteria will be read in detail and the final decision to include in the review will be based on relevance and rigour of the paper, as described by Pawson *et al*.^33^ Only documents which make a substantial contribution to developing PTs will be assessed for rigour to ensure the quality of the data used to test theories. Regardless of rigour, all documents that make some contribution to the developing theories will be included. This will be discussed in the strengths and limitations section of any outputs disseminated from the research.

### Data extraction, analysis and synthesis

Data from all included documents will be extracted using a standard data collection table, including key characteristics such as setting, sample size and approach to research. Full-text documents will be uploaded to NVivo to facilitate the organisation and coding of data. Coding will involve extracting and attaching contributing sections of text to relevant PTs.

Data analysis will use a realist logic of analysis with the aim of using the extracted data to develop, refine or refute PTs in line with the evidence available. Data analysis will use deductive (led by IPTs), inductive (led by evidence emerging from the included documents) and retroductive (inferences made on interpretations of the data) approaches. Evidence will be synthesised across sources to develop, refine or refute PTs to provide a set of well-refined theories for testing with evidence from primary qualitative data.

### Primary data collection

The proposed study will undertake primary qualitative data collection to better understand the experiences of PEH and the knowledge, attitudes and experiences of those who provide services designed to support them in relation to trauma, EF, social functioning, health needs and trauma-informed approaches to care. Qualitative data will be collected from key stakeholders including professionals involved in the delivery of services to support people with experience of homelessness (n=15–20) and people with experience of homelessness who have previously engaged with services (n=15–20).

### Sampling and recruitment

The sample size (n=30–40) will be achievable and enable us to reach the right balance between achieving saturation and retaining analytical flexibility. Here, we are borrowing from the concept of theoretical saturation used in grounded theory, in that data will be collected until it no longer contributes to developing, refining or testing the IPTs. We include flexibility in our sample size, so that while we will be testing the theories initiated through the literature, we will also generate new theories from the data, in an inductive explanatory endeavour. This will include the purposeful seeking of divergent as well as convergent experiences, as is customary in realist analysis, in keeping with the most recent thinking on sampling for qualitative research.^35^ Sampling will take a purposeful sampling approach,^36^ supporting the selection of individuals who are proficient, knowledgeable, experienced and well informed on the phenomenon of interest.

To participate in this study, PEH must be aged 18 or over and have experienced repeated (more than four episodes totalling a minimum of 12 months) or chronic (more than 12 months) homelessness. Adverse experiences during childhood will not be applied as an inclusion criterion for this study. Participants will, however, be asked about their childhood and any experiences of trauma during the interview. This research adopts a broad definition of homelessness, including those living in unsuitable or unstable accommodation, in temporary or sheltered housing, sofa surfing and rough sleeping. Identification and recruitment of participants with experience of homelessness will be supported by our partner organisations. Initial contact will be made via gatekeepers at these organisations either in person via our networking events or email. Information sheets will be provided to gatekeepers to help them make an informed decision about supporting the research prior to any individual-level recruitment. PEH can opt to contact the research team themselves or leave contact details to allow the research team to contact them. Full informed consent will be sought from them prior to any data collection.

#### Professionals delivering services for PEH

Professionals delivering services for PEH will be sampled based on their knowledge and experience of working with PEH. We will seek to include participants from a wide range of services. Services will be sampled from our wider network of engaged partners (n=192 organisations). Opportunities to participate will be disseminated face to face through our networking events and via email across our wider distribution list.

Where appropriate, samples may be grown using snowballing techniques.

### Data collection

All data will be collected using semistructured interviews lasting approximately 1 hour. Participants will only be interviewed once, unless a follow-up interview is requested where the participant has further information they wish to share. Wherever possible, interviews will be conducted face to face with at least one member of the research team. Every effort will be made to accommodate specific requests, such as the gender of the interviewer, to ensure both the comfort and safety of participants and researchers. All interviews will be recorded on encrypted recorders and transcribed verbatim for analysis. At the conclusion of each interview, both participants and research staff will be provided with an opportunity for a debrief.

Specific details for each participant group are provided below.

#### People with experience of homelessness

PEH interviews will take place on the premises of the service through which they were recruited or on university premises depending on the preferences and needs of the participant. Participants will be advised that they may bring a support person with them if desired, and steps will be taken to ensure support staff are available following the interview, regardless of the location of the interview.

Interviews will cover topics such as education, employment, relationships, mental health, substance use and engagement with services, as well as experiences which relate specifically to EF such as time management, planning, organising and completing tasks, memory and emotion regulation. Participants will also be asked if they consider themselves to have experienced trauma, but no further details will be requested.

#### Professionals delivering services for PEH

Interviews with service providers and allied health professionals will take place on their own service premises where possible. Should another venue be preferred, interviews will be conducted at Northumbria University. Interviews with professionals from services for PEH will seek to understand current TIC approaches to service delivery, understanding of EF and its potential impacts on capabilities and behaviours when engaging in services, any adaptations that are made within the service and any resource or training needs around TIC.

### Data analysis and evidence synthesis

Interviews will be transcribed verbatim, and the files will be uploaded in the same NVivo database as the realist synthesis papers above. As before, PTs will form the unit of analysis, as we will take a deductive (led by IPTs), inductive (led by evidence emerging from the interviews, eg, on understanding of EF) and retroductive (inferences made on interpretations of the data) approaches to analysis.

Evidence from both published literature and interviews will be synthesised to further test and refine or refute developing PTs to provide comprehensive insight into the role of EF as a result of trauma in pathways into, experiences of and routes out of homelessness, including what works or does not in engagement with services, for who, in what circumstances and why. Further to this, we will consider how a broader approach to TIC, to include neurobiological considerations such as EF, may lead to better outcomes for PEH.

The whole analytical process will be iterative, in that findings from the primary data may lead to further searches of the literature, until we reach analytical saturation and are able to answer the research question in a rich and nuanced manner, drawing on transdisciplinary insights.

This project commenced in November 2024 and is scheduled to conclude in January 2026.

## Ethics and dissemination

This study has received ethics approval from the ethics committee at Northumbria University (Ref: Cooper 2024-7761-8214).

Anonymised findings from this research will be included in a range of academic outputs such as conference papers and presentations, published journal articles, podcasts and blogs. Further to this, findings will inform the development of a toolkit designed to support service providers in broadening approaches to TIC. Further funding will also be sought to continue this work, including development, piloting and testing of the toolkit.
