# Supplementary material for: Understanding trauma-impaired executive function and its impacts on homeless adults in the UK: a realist evaluative synthesis protocol
Source: BMJ Open. 2025 Jun 20;15(6):e093529. doi: 10.1136/bmjopen-2024-093529 (PMC12182018; doi:10.1136/bmjopen-2024-093529)
Supplement: Supplementary file 1 [file bmjopen-2024-093529.reviewer_comments.pdf]

PEER REVIEW HISTORY

BMJ Open publishes all reviews undertaken for accepted manuscripts. Reviewers are asked to complete a checklist review form and are provided with free text boxes to elaborate on their assessment. These free text comments are reproduced below.

ARTICLE DETAILS

Title (Provisional)

Understanding trauma impaired executive function and its impacts in homeless adults in the UK: a realist evaluative synthesis protocol

Authors

Cooper, Christina; Lhussier, Monique

VERSION 1 - REVIEW

**Reviewer**	**1**
**Name**	**Driessen, Martin**
**Affiliation**	**Universitätsklinik und Poliklinik für Psychiatrie Bern**
**Date**	**06-Feb-2025**
**COI**	**None**

In this qualitative approach the authors are constructing a link between childhood adverse experiences (CAE), disturbed executive functioning (EF), homelessness (PEH), user behaviors, and forms of conventional as well as trauma-focussed care. In addition, traumatic brain injuries as well as stroke are introduced with their impact on cognitive functioning and which may be also found in homeless peoplle. By interviewing some of those concerned as well as caregivers and representatives of the stakeholders the authors intend to understand the life course experiences of PEH, to understand lived experiences that may be underpinned by impaired EF, to understand how these experiences may intersect in cumulative and cyclical ways across the life course, to identify challenges resulting from experiences of EF impairments as a barrier to engagewith support services, to explore service providers' understanding of EF, to identify current approaches to TIC being implemented in services for PEH, to identify resource and training needs of services which intend to include TIC, to understand how services for people with acquired brain injury/stroke accommodate EF dysfunction, to develop a suite of training, tools, and resources for services.

The manuscript is interesting to read and focusses on a relevant problem in homeless people and improving services is urgent. I feel there is a lot if work included in planning the project.

However, I see some serious problems with this paper.

1. One major problem is putting together a great amount of completely different dimensions: previous events (CAE), neuropsychology (EF), also acquired brain dysfunctiosn after injuries and stroke, a social dimension (homelessness), (non)using behavior regarding servives, as well as the structure of services themselves. Each of these relations is highly complex and would have to be observed or investigated carefully.

2. Even if the authors intend to refer to previous research regarding the link between CAE and EF they must choose the sample carefully, i.e. it must be assessed that participants have a history of CAE and EF disturbances but no history of brain injury or stroke (which are frequent in homeless people). The authors may also study such a group but separately. And how should those who work in services be able to differentiate between these groups. Thus, I fear that results of a study as it stands now, remain unclear.

3. I miss a rationale for the sample sizes of the different groups of participants.

4. I the attached replies to previous reviewers, work packages are noted. However, I dot find them in the manuscript.

VERSION 1 - AUTHOR RESPONSE

Response to reviewer

Reviewer's comment	Authors' response
One major problem is putting together a great amount of completely different dimensions: previous events (CAE), neuropsychology (EF), also acquired brain dysfunctions after injuries and stroke, a social dimension (homelessness), (non)using behavior regarding services, as well as the structure of services themselves. Each of these relations is highly complex and would have to be observed or investigated carefully	Thank you for such insightful comment. We have amended the protocol to remove the brain injuries and stroke dimensions.<br><br>However, we believe that examining this diverse set of constructs within one population group (people who have experienced homelessness) is precisely the key contribution that our paper makes. This has been now clarified further through reducing the number of objectives, and simplifying the text wherever possible. We have amended the introduction to make our thinking more explicit.
Even if the authors intend to refer to previous research regarding the link between CAE and EF they must choose the sample carefully, i.e. it must be assessed that participants have a history of CAE and EF disturbances but no history of brain injury or stroke (which are frequent in homeless people). The authors may also study such a group but	This is a very fair point, thank you for raising it. The premise of the study is that the link that is already well established between ACEs (Adverse Childhood Experiences) and executive dysfunction in childhood, continues in adulthood and might set people on a course that is more likely to include homelessness. If people subsequently

separately. And how should those who work in services be able to differentiate between these groups. Thus, I fear that results of a study as it stands now, remain unclear.	developed an acquired brain injury or a stroke, this would only compound the early executive dysfunction, as would, indeed, drug or alcohol addictions, for example. As we are not trying to establish a straightforward causal relationship between ACEs and homelessness, subsequent events compounding executive dysfunction are not seen as confounding factors, but as part of the qualitative experiences we are exploring. Consistent with realist inquiry, we are interested in a generative (rather than successionist) model of causation, whereby various experiences combine to explain an observed pattern of outcomes – in our case, the lack of engagement of people who experience homelessness with services that might help them. The study aims to, based on the established literature and empirical data, explain these relationships in a novel way, which has face validity, and therefore resonance with practice. As well as now excluding physical brain injuries from our samples in order to streamline the protocol, we have now expanded on this throughout the text.
I miss a rationale for the sample sizes of the different groups of participants.	We have now added a paragraph to this effect, under Sampling and Recruitment (p7).
I the attached replies to previous reviewers, work packages are noted. However, I dot find them in the manuscript	When submitting this protocol for review we submitted the original funding application, reviewers comments and response to reviewers as part of the submission as this can sometimes alleviate the need for further peer review. This is the documents you are seeing. The project detailed in the proposal is one of 5 work packages we will be conducting. While findings from this project (work package one in the bid) may be used to inform other work packages it has merit as a standalone research project in and of itself. To save confusion and conform with word count the decision was made to publish the protocol for this work package alone initially given its novelty and the complexity of the topic.
